# Are you more risk-seeking when helping others? Effects of situational urgency and peer presence on prosocial risky behavior

**DOI:** 10.3389/fpsyg.2023.1036624

**Published:** 2023-02-27

**Authors:** Changlin Liu, Xiao Xiao, Qiao Pi, Qianbao Tan, Youlong Zhan

**Affiliations:** ^1^Department of Psychology, Hunan University of Science and Technology, Xiangtan, China; ^2^Mental Health Education Center, Hunan First Normal University, Changsha, China

**Keywords:** helping dilemmas, peer presence, risk preferences, prosocial risky behavior, situational urgency, social preferences

## Abstract

**Introduction:**

Prosocial risky behavior (PRB) proposes that individuals take risks for others’ benefits or social welfare, and that this may involve trade-offs between risk and social preferences. However, little is known about the underlying cognitive mechanisms of risk-seeking or aversion during PRB.

**Methods:**

This study adopted the dilemma-priming paradigm to examine the interaction between the risk levels of personal cost and situational urgency on PRB (Experiment 1, *N*  = 88), and it further uncovered the modulation of the risk levels of failure (Experiment 2, *N*  = 65) and peer presence (Experiment 3, *N*  = 80) when helping others.

**Results:**

In Experiment 1, the participants involved in risky dilemmas made more altruistic choices for strangers in urgent situations compared to those for strangers in non-urgent situations. However, increasing the risk levels of personal cost decreased the frequencies of help offered to strangers in urgent situations. Experiment 2 further established that, similar to the risk of personal cost, increasing the risk levels of failure when helping others also decreased the frequencies of help offered to strangers in urgent situations. Furthermore, in dilemmas involving a low-risk personal cost, Experiment 3 showed that peer presence encouraged the participants to make more altruistic choices when providing help to strangers in non-urgent situations.

**Discussion:**

Individuals demonstrate obvious risk-seeking behavior when helping others and that both non-urgent situations and peer presence weaken the effect of increased risk aversion on PRB in a limited manner.

## 1. Introduction

In health-or life-threatening situations, the motivation to protect ourselves may conflict with that to protect others (Vieira et al., 2020). For example, in the global pandemic of COVID-19, the desire to avoid being infected by the virus may keep healthcare workers from reaching the front lines of the epidemic to save infected patients. Decisions to help others in such risky situations or crises differ from common everyday helping, in which individuals require the integration of two highly salient cues—the risks or dangers to ourselves, and the suffering or harms of others in need. To date, numerous studies involving the cognitive mechanism of human prosocial behaviors have mainly focused on the latter (i.e., on how empathy for others’ suffering or harm induced altruistic motivation). However, the role of concurrent defensive responses to risks or dangers has been neglected. Recently, Telzer and colleagues proposed prosocial risky behavior (PRB) to describe and explain this special behavior, of which the original intent was for the benefit of others or social welfare at the cost of assuming certain unknown risk losses for themselves (e.g., physical, emotional, or social; Do et al., 2017). This distinguishes prosocial risky behavior from simple prosocial and risky behaviors. For simple risky behaviors, the completion of the behavior does not have the necessary benefit to others or to society, but only poses a risk to oneself or to other potentially relevant individuals. For simple prosocial behaviors, the completion of the behavior directly benefits others or society, but does not pose an unknown or uncertain risk to oneself. Although some simple prosocial behaviors may incur personal costs or losses (e.g., donating time or money to a charity), such costs or losses are usually known, whereas the risk-related costs borne by individuals in prosocial risky behaviors are unknown or uncertain. Thus, the uniqueness of prosocial risky behavior, compared to simple prosocial behavior, lies in the unknown nature of the cost of the behavior.

PRB is usually observed in real-life and experimental laboratory situations, whereby individuals assume specific risk costs, such as monetary loss (Greening et al., 2014), immediate physical harm (Hein et al., 2010; Vieira et al., 2020), or hypothetical effective and socially negative outcomes (Saito, 2013; Zhan et al., 2018), for others’ benefits (De Waal and Preston, 2017). For example, participants involved in such studies were observed to take a certain risk of monetary loss to achieve enhanced procedural and distributive fairness between themselves and strangers (Brennan et al., 2008; Güth et al., 2008; Karni et al., 2008; Krawczyk and Le Lec, 2010; Gross et al., 2020). Therefore, in addition to the ability to empathize with others under various threats or dangers, i.e., social preferences (Zhang et al., 2019; Vieira et al., 2020), the extent to which risk factors affects prosocial outcomes has also been associated with individual differences, such as psychological responses to risk sources, i.e., risk preferences (Schweda et al., 2019; Petko et al., 2021). However, although these studies show that prosocial risky decisions are influenced by the risk levels of personal cost, the way in which the potential mechanism of this influence and the way in which it modulates social preferences remains unclear (Crockett et al., 2014; Volz et al., 2017). Additionally, induced stress paradigms are not entirely comparable to real-life threatening or risky scenarios in which immediate physical or social risk is associated with providing help.

Social preferences reflect the tendency of individuals to focus on the benefits of others or the welfare of society (Deng et al., 2016). Situational urgency is one of the key factors affecting individuals’ social preferences when making prosocial decisions, and it involves the degree of urgency and conflict caused by the empathy toward other individuals exposed to threats, dangers, suffering, or harm (Forgas and Cromer, 2004; Zhang et al., 2021). Previous studies have suggested that when individuals are in situations that require urgent help, they can perceive significant levels of internal conflict and realize active avoidance by rapidly activating and coordinating with the neural circuits responsible for specific defense behaviors toward dangers or threats, and thus, they can engage in risk avoidance behaviors by ignoring the suffering and harm of others (Fanselow and Lester, 1988; Blanchard and Blanchard, 1990). However, recent studies have reported that defensive states driving active escape from immediate danger may also encourage people to ignore the risk of personal costs and offer additional help to others in threat or danger (Rand et al., 2014; Rickenbacher et al., 2017). For example, Vieira et al. (2020) reported that regardless of how likely participants were to receive painful shock, imminent threats, compared to distant threats, inclined them to provide additional defensive help to others, and this promotion was more obvious among individuals with higher scores in empathetic concern for the suffering of others. These findings support the notion that situational urgency may engage processes implicated in the motivation to provide care among humans, despite taking a certain risk of benefit loss. However, little is known regarding the way in which PRB is influenced by the interaction between the risk of personal cost and situational urgency.

Generally, the different types of risks resulting from helping others are complex and interactive, and they usually include the two types of risks associated with both the personal cost and the failure to help others (Zlatev et al., 2019; Gross et al., 2020). For example, the higher the risk levels of the failure to help others, the more likely individuals are to avoid risk by activating and rapidly coordinating specific defense behaviors, such as escape (Fanselow and Lester, 1988; Blanchard and Blanchard, 1990). However, other studies have demonstrated that when people share the risk of monetary loss or physical harm with their partners, they are more willing to help their partners despite facing high levels of personal cost (Hein et al., 2010; FeldmanHall et al., 2012; Bixter and Luhmann, 2014; Vieira et al., 2020). Therefore, when the helping dilemma involves different levels and types of risk, i.e., the risk of personal cost and the risk of failure when helping others, it is imperative to determine the way in which individuals trade off and integrate risky information with prosocial information in the case of PRB.

Additionally, previous studies have demonstrated that the presence of peers encourages risk-seeking tendencies (Chein et al., 2011; Logue et al., 2014; Smith et al., 2014; Tian et al., 2018) and prosocial tendencies among different individuals (Kurzban et al., 2007; Bereczkei et al., 2015; Xin et al., 2016; Wang and Dai, 2020). The presence of peers makes the individuals involved highly sensitive to social information, i.e., evaluation and feedback from others, and it further encourages them to neglect risk information and pay significant attention to potentially larger benefits (Gardner and Steinberg, 2005; Shulman and Cauffman, 2013). Moreover, recent studies on moral decision-making have reported that participants make more altruistic choices in the presence of peers because they show higher levels of reputational concern (Minwoo et al., 2018; Zhan et al., 2020a,b). Therefore, these findings suggest that peer presence can influence individuals’ perceptions and preferences toward risk-related information when making risky decisions and that situational urgency affect prosocial decisions. However, it is crucial to determine the way in which peer presence modulates the interaction between the risk of personal cost and situational urgency when making decisions associated with PRB.

In conclusion, some previous studies have regarded prosocial risky behavior as simple prosocial behavior, focusing primarily on the influence of risk factors. Other researchers regarded prosocial risky behavior as simple risk-taking behavior, mainly focusing on prosocial motivation’s influence. Prosocial risky behavior involves the integrated processing of individual risk and social preferences. In this study, we aimed to examine the influence of situational factors such as levels and sources of risk on PRB and further explain the underlying trade-offs and integration mechanisms associated with the social and risk preferences. Therefore, we first examined whether PRB was affected by the interaction between the risk of personal cost caused by helping others and situational urgency (Experiment 1). Vieira et al. (2020) reported that situational urgency encouraged participants to engage in additional helping behaviors regardless of the risk levels of undergoing shock following a decision to help. Therefore, we predicted that when participants assumed the risk of personal cost, this altruistic tendency in urgent situations would only be observed in situations with low-risk levels of personal cost, compared to non-urgent situations. However, compared to non-urgent situations, increased risk levels of personal cost would have a stronger weakening effect on individual PRB in urgent situations owing to increased levels of risk aversion. Our second question involved the impact of risk types, i.e., the risk of personal cost and the risk of failure when helping others, when making decisions to help strangers under different degrees of situational urgency (Experiment 2). Relevant studies have suggested that the risks caused by helping others are complex and interactive (Zlatev et al., 2019; Gross et al., 2020). Therefore, we predicted that the differences in the frequencies for providing help in urgent and non-urgent situations would be synergistically modulated by the levels of the two types of risks as they pertain to PRB. Our third and final question involved the influence of peer presence on risk and situational urgency, as it pertains to PRB (Experiment 3). Previous studies have demonstrated that peer presence enhances risk-seeking tendencies when making decisions (Smith et al., 2014; Tian et al., 2018) and altruistic tendencies when making social decisions (Xin et al., 2016; Wang and Dai, 2020). Therefore, we hypothesized that peer presence can increase the frequency of PRB-related help by focusing less on the risk levels of personal cost. However, this effect would be increasingly robust in the case of non-urgent situations. Additionally, we reported the measures and manipulations of all the variables involved in this study.

## 2. Experiment 1

Experiment 1 aimed to examine the infulence of risk levels of personal cost and situational urgency on PRB. We hypothesized the following: (1) compared to non-urgent situations, participants would make more altruistic decisions toward strangers in urgent situations, (2) the frequencies of providing help would be higher in situations involving low-risk levels of personal cost, (3) and increased risk levels of personal cost would have a stronger weakening effect on the frequencies of help provided to strangers in urgent situations compared to those in non-urgent situations owing to increased levels of risk aversion.

### 2.1. Methods

#### 2.1.1. Participants

G* Power 3.1 software was used to calculate the sample size (Franz et al., 2007), and the results showed that this study needed to recruit at least 30 participants to ensure sufficient test efficacy (effect = 0.90) under the premise of a medium effect size (*f* = 0.30) according to the study conducted by Vieira et al. (2020). Therefore, 88 college students (44 females) were recruited to participate in this study. All the participants were right-handed and had normal or corrected-to-normal vision. The experiment was conducted in accordance with the Declaration of Helsinki and was also approved by the Ethics Committee of Hunan University of Science and Technology. After understanding the experiment fully, each participant signed an informed consent form.

### 2.2. Materials and procedure

#### 2.2.1. Risky helping dilemmas

Twenty everyday helping dilemmas (see Supplementary Table 1) were cited from previous studies on moral decision-making to measure PRB among the participants (Zhan et al., 2018, 2019, 2020a,b). Meanwhile, according to the situational urgency toward participants in these dilemmas, 20 dilemmas were divided into two types: urgent and non-urgent situations, each with 10 dilemmas. Additionally, to effectively measure the risk and social preferences associated with PRB, 20 helping dilemmas were adapted to produce the two types of high-risk and low-risk levels of personal cost toward oneself. According to previous studies on risk decisions (Sun et al., 2009; Song et al., 2012), risk levels of personal cost were operated at 95%, i.e., high-risk level, and 5%, i.e., low-risk level, probability that helping others would result in a loss of self-benefit, i.e., time, health, and safety. Therefore, there were four types of dilemmas: high-risk levels of personal cost and urgent situations toward participants (HU), low-risk levels of personal cost and urgent situations toward participants (LU), high-risk levels of personal cost and non-urgent situations toward participants (HN), and low-risk levels of personal cost and non-urgent situations toward participants (LN), each with 5 dilemmas. Specific examples of these dilemmas are listed in Table 1.

**Table 1:** The sequence of a single trial in the prosocial risky behavior task.

	High-risk levels of personal cost	Low-risk levels of personal cost
Urgent situation	When you are on the way to the postgraduate entrance examination, someone is hit by a car. He is in dire need of your help to send him to the hospital for treatment	When you are on the way to the postgraduate entrance examination, someone is hit by a car. He is in dire need of your help to send him to the hospital for treatment
	F: If you help, he will get medical attention, but you will be 95% likely to miss the exam	F: If you help, he will get medical attention, but you will be 5% likely to miss the exam
	J: Without help, he will be in danger of not being treated in time, but you will take the exam successfully	J: Without help, he will be in danger of not being treated in time, but you will take the exam successfully
	**(HU)**	**(LU)**
Non-urgent situation	Someone is a poor student in the village, who is in urgent need of a large number of tuition fees after he is admitted to the university. You are a poverty alleviation village cadre. The village has just applied for a sum of money to repair the well, and the villagers are excited, but at this time he asks you for help	Someone is a poor student in the village, who is in urgent need of a large number of tuition fees after he is admitted to the university. You are a poverty alleviation village cadre. The village has just applied for a sum of money to repair the well, and the villagers are excited, but at this time he asks you for help
	F: If you help, his tuition problem will be solved, but you will be **95%** likely to delay the well repair work and be blamed by the villagers	F: If you help, his tuition problem will be solved, but you will be **5%** likely to delay the well repair work and be blamed by the villagers
	J: Without help, he will not be able to go to school and thus abandon his studies, but the task will be completed successfully	J: Without help, he will not be able to go to school and thus abandon his studies, but the task will be completed successfully
	**(HN)**	**(LN)**

Specifically, each dilemma describes a conflict situation in which protagonists must sacrifice their benefits to help strangers in urgent or non-urgent situations. Each dilemma comprises a scenario and two options. The scenario describes a situation in which the protagonist desperately needs help (involving urgent or non-urgent situations) while one is conducting an important deed, and the individual must decide whether to aid the person and give up doing their important deed. The two options describe the outcomes of the decision to help or not: one option describes an altruistic decision that one is going to give up their important goal to help the other person (involving high-risk or low-risk levels of personal cost), and the other option describes an egoistic decision that one is going to keep doing their important task, thereby ignoring the other person. Participants were required to choose between the two options. The numbers and familiarities of risky helping dilemmas were controlled and balanced between participants.

Meanwhile, before the experiment, 30 participants were recruited to complete the 7-point rating scale item (“How urgently do you feel that the stranger is in a threatening situation?” 1 = not at all urgent, 7 = extremely urgent) to assess the degree of threat urgency toward the stranger in every helping dilemma. The results showed that the non-urgent dilemmas (*M* = 3.60 ± 0.15) were significantly less than the urgent dilemmas (*M* = 4.48 ± 0.17, *p* < 0.001), *F*_(1, 58)_ = 73.80, *p* < 0.001, *η*_p_^2^ = 0.56. This result showed that situational urgency was effective.

#### 2.2.2. Procedure

**Figure 1:**
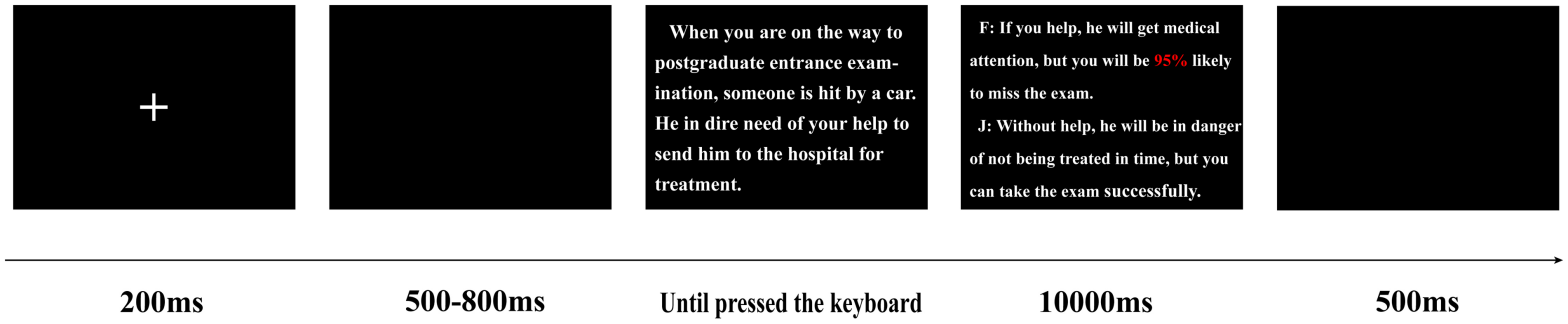
The sequence of a single trial in the prosocial risky behavior task.

The sequence of the risky helping dilemma task is shown in Figure 1. To improve the participants’ attention, a fixation cross was presented for 200 ms, followed by a blank screen for 500–800 ms (the exact time was random). The text of the scenario was then presented for an unlimited time until the participant pressed a button on the keyboard. The two behavioral options (helping the person or not helping the person) were then presented for 10,000 ms, and the participants were asked to decide between the two options by pressing one of two keys quickly (the key associated with each option was balanced between participants). Finally, a blank screen was presented for 500 ms. All stimulus presentations were accomplished using the E-prime software (Psychology Software Tools, Pittsburgh, PA, United States).

### 2.3. Data statistics and analysis

Experiment 1 used a 2 (risk levels of personal cost: high-risk levels vs. low-risk levels) × 2 (degrees of situational urgency: urgent situations vs. non-urgent situations) within-subject design. We recorded the frequencies of helping choices during prosocial risky helping tasks. ANOVA was used to analyze the effect of the two within-subject factors on this dependent variable. SPSS software (version 20.0) was used to perform the statistical analysis of all the data.

### 2.4. Results

The overall frequencies of the trials in which participants selected help were 0.65 (SD = 0.18). A one-sample *t*-test indicated that the average helping frequencie was significantly higher than the 50% chance, *t*_(87)_ = 7.58, *p* < 0.001, thereby suggesting that participants tended to help the protagonists in risky dilemmas. Moreover, the main effect of the degrees of situational urgency was significant, *F*_(1, 87)_ = 13.61, *p* < 0.001, *η*_p_^2^ = 0.14, thereby suggesting that participants made more altruistic choices for strangers in non-urgent situations (*M* = 0.68, SD = 0.21) than for strangers in urgent situations (*M* = 0.61, SD = 0.20). The main effect of the risk level of personal cost was significant, *F*_(1, 87)_ = 81.14, *p* < 0.001, *η*_p_^2^ = 0.48, thereby indicating that participants made more altruistic choices in situations involving low-risk levels (*M* = 0.74, SD = 0.21) compared to those involving high-risk levels (*M* = 0.55, SD = 0.20) of personal cost.

**Figure 2:**
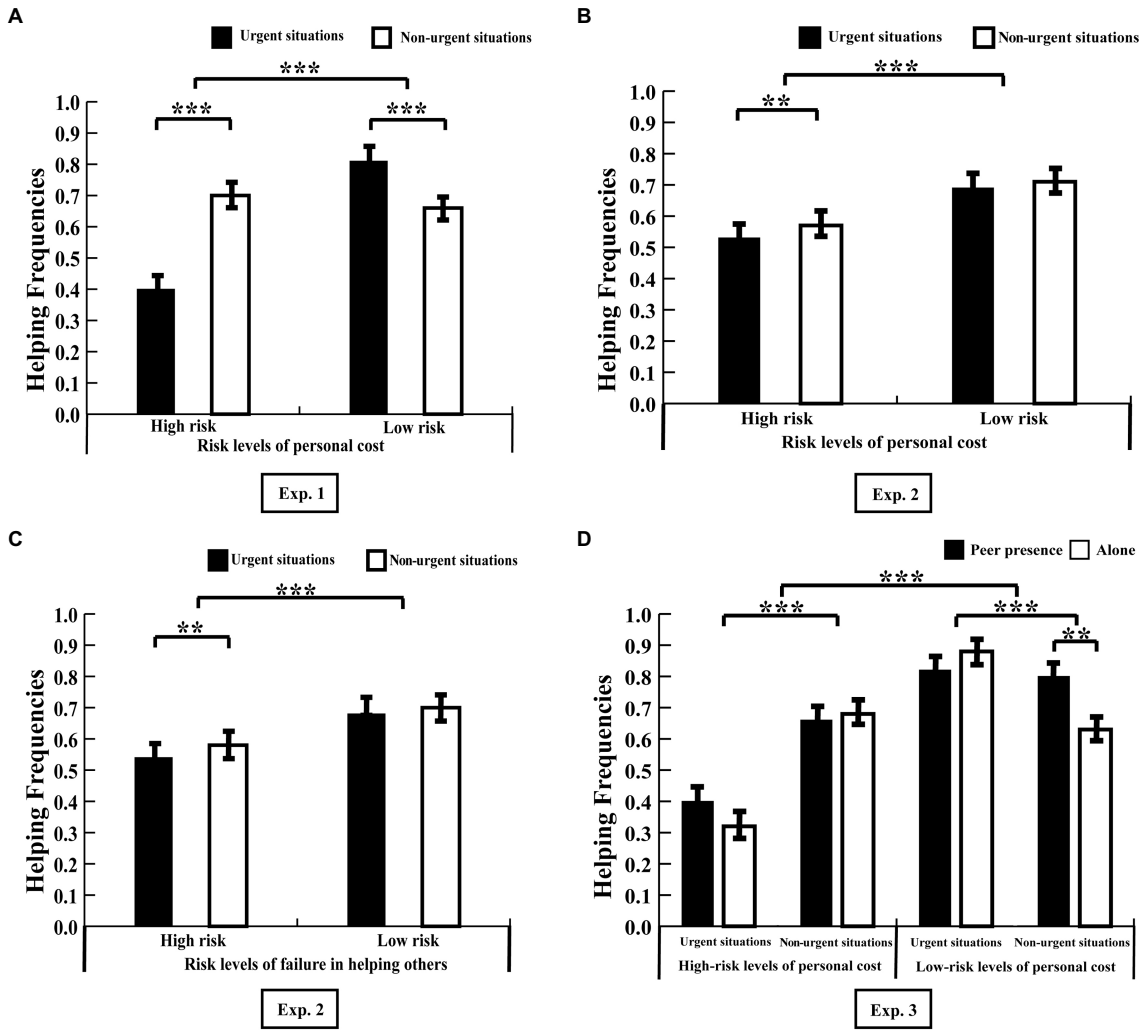
Helping frequencies under each condition in Experiment 1 **(A)**, Experiment 2 **(B,C)**, and Experiment 3 **(D)**. Error bars were drawn from the standard deviation and reflected 95% confidence intervals. **p < 0.01, ***p < 0.001.

The interaction between them was significant: *F*_(1, 87)_ = 107.45, *p* < 0.001, *η*_p_^2^ = 0.55. Further, through simple effect analysis, we established that the participants made more altruistic choices for strangers in urgent situations (*M* = 0.81, SD = 0.25) as compared to those in non-urgent situations (*M* = 0.66, SD = 0.25) in situations with low-risk levels of personal cost, *F*_(1, 87)_ = 102.21, *p* < 0.001. However, participants made less helpful choices for strangers in urgent situations (*M* = 0.41, SD = 0.25) compared to those in non-urgent situations (*M* = 0.70, SD = 0.24) or in situations with a high-risk level of personal cost, *F*_(1, 87)_ = 24.72, *p* < 0.001 (Figure 2A).

Additionally, we calculated the risk-taking tendencies using the helping frequencies of high-risk levels minus those of low-risk levels of personal cost in different urgent situations, which indexed the weakening effect of the increased risk levels of personal cost on social preferences. The results showed that risk-seeking tendencies in urgent situations (*M* = −0.41, SD = 0.30) were lower than those in non-urgent situations (*M* = 0.04, SD = 0.25), *t*_(87)_ = −10.37, *p* < 0.001.

### 2.5. Discussion

Through Experiment 1, we established that participants generally showed obvious risk-seeking tendencies during PRB, thereby indicating that people made more helpful decisions (more than 50% chance) in risky situations. This risk-seeking tendency in this study is consistent with that reported in previous studies, indicating that people made highly prosocial decisions for others’ benefits or welfare despite taking the risk of personal cost (Zhan et al., 2019, 2020a,b; Gross et al., 2020; Vieira et al., 2020). However, the weakening effect of the increased risk levels of personal cost on social preferences in urgent situations were stronger compared to those in non-urgent situations. This finding was in line with the findings of previous studies, which reported that higher levels of frequent helping decisions under distant threats suggested that defensive states associated with the engagement of slower and more flexible decision processes facilitated helping behavior in urgent situations (Buchanan and Preston, 2014). These findings supported our hypotheses for Experiment 1, which suggested that individuals’ social preferences caused by the degree of situational urgency during PRB were modulated by the risk preferences caused by the risk levels of personal cost. However, the different types of risks caused by situational factors are complex and interactive (Gross et al., 2020). Previous studies have suggested that the risk levels of failure to help others may also impact PRB (Zlatev et al., 2019; Gross et al., 2020). Therefore, Experiment 2 further explored the way in which the two types of risk, i.e., the risk of personal cost and the risk of failure in helping others, synergistically modulate the impact of situational urgency on PRB.

## 3. Experiment 2

Experiment 2 aimed to further examine the way in which both levels of risk, i.e., the risk of personal cost and the risk of failure in helping others, affected the frequencies of providing help to strangers in urgent and non-urgent situations. We hypothesized the following: (1) owing to the increased risk levels associated with the two types of risks, the frequencies of the decisions to provide help to strangers experiencing threats would decrease and (2) the difference between high-risk and low-risk levels of the personal cost would be only observed in situations involving low risk but not at high-risk levels of the failure to help others.

### 3.1. Methods

#### 3.1.1. Participants

Similar to Experiment 1, G* Power 3.1 software was used to calculate the sample size, and the results showed that this study needed to recruit at least 30 participants to ensure sufficient test efficacy (effect = 0.90) under the premise of a medium effect size (*f* = 0.30). Therefore, 65 college students (33 females) were recruited to participate in Experiment 2. The experiment was conducted in accordance with the Declaration of Helsinki and was also approved by the Ethics Committee of Hunan University of Science and Technology. After understanding the experiment fully, each participant signed an informed consent form.

### 3.2. Materials and procedure

#### 3.2.1. Operation of failure in helping others

Based on Experiment 1, eighty everyday helping dilemmas were cited from previous studies on moral decision-making to measure PRB among the participants (Zhan et al., 2018, 2019, 2020a,b). Meanwhile, according to the situational urgency toward participants in these dilemmas, 80 dilemmas were divided into two types: urgent and non-urgent situations, each with 40 dilemmas. Urgent and non-urgent dilemmas added the operation of high-risk and low-risk levels of the failure to help others, namely, a 95% or 5% chance of the failure to help others. Therefore, there were four types of dilemmas involving different levels and types of risks: high-risk levels of personal cost and high-risk levels of the failure to help others (HH), high-risk levels of personal cost and low-risk levels of the failure to help others (HL), low-risk levels of personal cost and high-risk levels of the failure to help others (LH), low-risk levels of personal cost, and low-risk levels of the failure to help others (LL), each with 10 urgent dilemmas and 10 non-urgent dilemmas. For example, when you are on your way to a postgraduate entrance examination, someone else is hit by a car. The victim is in dire need of your help to send him to the hospital for treatment. Option F: If you help them, you will be 95% likely to miss the exam and 95% likely to abortively help others. Option J: Without help, they will be in danger of not being treated in time, but you will take the exam successfully. Participants were required to choose between the two options. The numbers and familiarities of risky helping dilemmas were controlled and balanced between participants.

#### 3.2.2. Procedure

**Figure 3:**
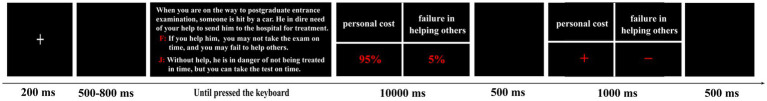
The sequence of a single trial during PRB under different levels and types of risks.

The sequence of the risky helping dilemma tasks is shown in Figure 3. To improve the participants’ attention, a fixation cross was presented for 200 ms, followed by a black screen for 500–800 ms (the exact time was random). The text of the scenario was then presented for an unlimited time until the participant pressed a button on the keyboard. The two behavioral options (to help the person or to not help the person) were then presented for 10,000 ms, and the participants were asked to decide between the two options by pressing one of two keys quickly (the key associated with each option was balanced between participants). Next, after a black screen was presented for 500 ms, the feedback of outcome about the helping decision was presented for 1,000 ms, thereby showing that “+” represents the outcome of self-benefit without loss or success when helping others and “−” represents the outcome of personal cost or failure when helping others. Finally, a black screen was presented for 500 ms. All stimuli presentations were accomplished using E-prime software (Psychology Software Tools, Pittsburgh, PA, United States).

### 3.3. Data statistics and analysis

Experiment 2 used a 2 (risk levels of personal cost: high-risk levels vs. low-risk levels) × 2 (degree of situational urgency: urgent situations vs. non-urgent situations) × 2 (risk levels of failure to help others: high-risk levels vs. low-risk levels) within-subject design. ANOVA was used to analyze the way in which the dependent variable (i.e., help frequencies) was affected by the three within-subject factors. SPSS software (version 20.0) was used to perform the statistical analysis of all the data.

### 3.4. Results

The overall frequencies of trials in which participants selected help were 0.63 (SD = 0.12). A one-sample *t*-test indicated that the average helping frequencie was significantly higher than the 50% chance (*t*_(64)_ = 8.36, *p* < 0.001), thereby suggesting that participants tended to decide to help protagonists experiencing threats. Moreover, the main effect of the degree of situational urgency was significant, *F*_(1, 64)_ = 6.98, *p* = 0.01, *η*_p_^2^ = 0.10, thereby suggesting that participants made more altruistic choices toward strangers in non-urgent situations (*M* = 0.64, SD = 0.13) compared to those in urgent situations (*M* = 0.61, SD = 0.13). Meanwhile, the main effect of the risk level of personal cost was significant, *F*_(1, 64)_ = 26.38, *p* < 0.001, *η*_p_^2^ = 0.29, thereby indicating that help frequencies in situations with low levels of personal cost (*M* = 0.70, SD = 0.13) were significantly higher than those in situations involving high levels of personal cost (*M* = 0.55, SD = 0.21). Moreover, the main effect of the risk level of failure when helping others was significant, *F*_(1, 64)_ = 41.11, *p* < 0.001, *η*_p_^2^ = 0.39, thereby showing that help frequencies in situations involving low risks of the failure to help others (*M* = 0.69, SD = 0.13) were significantly higher than those in situations involving a high risk of failure to help others (*M* = 0.56, SD = 0.16).

The interaction between the risk levels of personal cost and the degrees of situational urgency was significant, *F*_(1, 64)_ = 4.92, *p* = 0.03, *η*_p_^2^ = 0.07. Through further simple effect analysis, we established that in situations with high levels of personal cost, the participants made more altruistic choices toward strangers in non-urgent situations (*M* = 0.57, SD = 0.22) compared to those in urgent situations (*M* = 0.53, SD = 0.21), *F*_(1, 64)_ = 10.76, *p* = 0.002. However, this difference was not significant in situations with low-risk levels of personal cost (urgent situations: *M* = 0.69, SD = 0.14; non-urgent situations: *M* = 0.71, SD = 0.14), *F*_(1, 64)_ = 2.57, *p* = 0.11 (Figure 2B).

The interaction between the risk levels of the failure to help others and the degrees of situational urgency was significant, *F*_(1, 64)_ = 4.98, *p* = 0.03, *η*_p_^2^ = 0.07. Through simple effect analysis, we established that, in situations involving high-risk levels of the failure to help others, the participants made more altruistic choices toward strangers in non-urgent situations (*M* = 0.58, SD = 0.18) compared to those in urgent situations (*M* = 0.54, SD = 0.17), *F*_(1, 64)_ = 9.93, *p* = 0.002. However, this difference was not significant in situations involving low-risk levels of personal cost (urgent situations: *M* = 0.68, SD = 0.14; non-urgent situations: *M* = 0.70, SD = 0.14), *F*_(1, 64)_ = 2.78, *p* = 0.10 (Figure 2C). However, the interaction between the three independent variables was not significant, *F*_(1, 64)_ = 0.24, *p* = 0.63, *η*p^2^ = 0.01.

Additionally, the risk-taking tendencies resulting from the risk of personal cost associated with helping strangers in urgent situations (*M* = −0.17, SD = 0.25) was lower than that in non-urgent situations (*M* = −0.15, SD = 0.25), *t*_(64)_ = −2.22, *p* = 0.03. Moreover, the risk-seeking tendencies resulting from the risk of failure to help strangers in urgent situations (*M* = −0.15, SD = 0.18) was lower than that in non-urgent situations (*M* = −0.13, SD = 0.18), *t*_(64)_ = −2.23, *p* = 0.03.

### 3.5. Discussion

Through Experiment 2, we further established that, similar to the risk of personal cost, increasing risk levels of the failure to help others also decreased individuals’ frequencies of help provided to strangers experiencing threats. This is consistent with previous studies showing that people are generally risk-averse when faced with high-risk levels of the failure to help others (Zhan et al., 2019, 2020a,b). Moreover, compared to non-urgent situations, the weakening effect of the increased risk levels of both personal cost and the failure to help others on social preferences in urgent situations were stronger. This was supported by a relevant study indicating that increasing risk levels might be a stronger predictor of PRB (Gross et al., 2020). However, the interaction between the two types of risks was not observed in the frequencies of the help provided. We speculated that different types of risks might independently modulate the effect of situational urgency on PRB. Therefore, increased risk levels of either personal cost or the failure to help others alone weakened individuals’ altruistic preferences toward strangers in urgent situations. However, it will be interesting to explore whether there exists a method that can be used to better restrain the negative impact of the obvious risk aversion caused by helping others during PRB. Previous studies have demonstrated that peer presence promotes both risk-taking and altruistic tendencies during economic and social decision-making (Bhm and Regner, 2013; Shulman and Cauffman, 2013). Therefore, Experiment 3 further explored the way in which peer presence modulated the interaction effect between the risk levels of personal cost and situational urgency on PRB.

## 4. Experiment 3

Experiment 3 examined whether peer presence promoted altruistic tendencies during PRB in different urgent situations when facing different risk levels of personal cost. We hypothesized the following: (1) compared to the alone condition, participants under the peer presence condition would make more altruistic choices toward strangers experiencing threats and (2) this promotion effect would be more robust in situations involving low-risk levels (vs. high-risk levels) of personal cost under urgent (vs. non-urgent) situations.

### 4.1. Methods

#### 4.1.1. Participants

Similar to Experiment 1, G* Power 3.1 software was used to calculate the sample size, and the results showed that this study needed to recruit at least 66 participants to ensure sufficient main effect test efficacy of the between-subject factor and at least 40 participants to ensure sufficient interaction effect test efficacy (effect = 0.90) under the premise of medium effect size (*f* = 0.30). Therefore, 80 college students (40 females) were recruited to participate in Experiment 3. Participants were randomly divided into the peer presence condition (*n* = 40, female = 20) and the alone condition (*n* = 40, female = 20). The experiment was conducted in accordance with the Declaration of Helsinki and was also approved by the Ethics Committee of Hunan University of Science and Technology. After understanding the experiment fully, each participant signed an informed consent form.

### 4.2. Materials and procedure

Each participant was asked to participate in this experiment with two friends of the same gender. Participants in the alone condition were instructed to perform the risky helping dilemma task alone, whereas the other two friends waited outside the sitting room. However, participants in the peer presence condition performed the risky helping dilemmas task in the same room as the two peers, and they were allowed to communicate with each other (Gardner and Steinberg, 2005; Defoe et al., 2020). The participants between the two conditions performed the same risky helping dilemma tasks as those in Experiment 1.

### 4.3. Data statistics and analysis

In Experiment 3, we used a 2 (risk levels of personal cost: high-risk levels vs. low-risk levels) × 2 (degree of situational urgency: urgent situations vs. non-urgent situations) × 2 (decision conditions: peer presence conditions vs. alone conditions) mixed design. ANOVA was used to analyze the way in which the dependent variable (i.e., help frequencies) was affected by both the within-subject variables (e.g., risk levels of personal cost and degrees of situational urgency) and the between-subject variable (e.g., decision conditions). SPSS software (version 20.0) was used to perform the statistical analysis of all the data.

### 4.4. Results

The overall frequencies of trials in which the frequencies of participants under the peer presence condition and the alone condition selected help were 0.67 (SD = 0.11) and 0.63 (SD = 0.15), respectively. The one-sample *t*-test indicated that the average helping frequencie was significantly higher than the 50% chance (peer presence condition: *t*_(39)_ = 9.84, *p* < 0.001; control condition: *t*_(39)_ = 5.50, *p* < 0.001), thereby suggesting that participants in the two conditions tended to decide to help the protagonists experiencing threats. Moreover, the main effect of the degree of situational urgency was significant, *F*_(1, 78)_ = 18.74, *p* < 0.001, *η*_p_^2^ = 0.19, thereby suggesting that participants made more altruistic choices toward strangers in non-urgent situations (*M* = 0.69, SD = 0.18) than those in urgent situations (*M* = 0.61, SD = 0.13). The main effect of the risk level of personal cost was significant, *F*_(1, 78)_ = 179.18, *p* < 0.001, *η*_p_^2^ = 0.70, thereby showing that help frequencies in situations involving low-risk levels of personal cost (*M* = 0.78, SD = 0.15) were significantly higher than those in situations involving high-risk levels of personal cost (*M* = 0.52, SD = 0.17).

The interaction between the three independent variables was significant, *F*_(1, 78)_ = 16.24, *p* < 0.001, *η*_p_^2^ = 0.17. Through further simple effect analysis, we established that in situations involving low levels of personal cost, participants in the peer presence condition made more altruistic choices toward helping strangers in non-urgent situations (*M* = 0.80, SD = 0.20) compared to participants in the alone condition (*M* = 0.63, SD = 0.26), *F*_(1, 78)_ = 10.01, *p* = 0.002. However, this difference between the two conditions was not significant in urgent situations (peer presence condition: *M* = 0.82, SD = 0.14; alone condition: *M* = 0.88, SD = 0.17), *F*_(1, 78)_ = 2.97, *p* = 0.09. Meanwhile, there were no other significant differences between the two conditions in situations involving high-risk levels of personal cost under urgent (peer presence condition: *M* = 0.40, SD = 0.17; alone condition: *M* = 0.32, SD = 0.21) and non-urgent (peer presence condition: *M* = 0.66, SD = 0.22; alone condition: *M* = 0.68, SD = 0.21) situations (*Fs* < 3.49, *ps* > 0.07, as shown in Figure 2D). Additionally, there were no other significant main or interaction effects (*Fs* < 2.46, *ps* > 0.12).

### 4.5. Discussion

Through Experiment 3, we further established that peer presence encouraged individuals to make more helpful decisions toward strangers experiencing threats. This result suggests that peer presence weakens individuals’ risk-aversion tendency to make more helpful decisions despite facing the risk of personal cost. This is consistent with previous studies reporting that peer presence promotes individuals’ risk-seeking tendencies, which in turn leads people to engage in PRB despite facing the risk of personal cost (Brechwald and Prinstein, 2011; Albert et al., 2013). People might adopt impression management strateges when facing prosocial risk situations. To gain acceptance and favor from their peers, college students under peer presence conditions might be more willing to take the risk of helping others than remain under the alone condition (Silva et al., 2016). However, this promotion effect of peer presence was only observed in non-urgent situations with low-risk levels of personal cost. In this study, there were no significant differences in the frequencies for providing help to strangers in urgent situations between peer presence and alone conditions as they pertain to situations involving high-risk levels of personal cost. The results suggest that individuals in the presence of peers fully weighed and integrated risk information and prosocial information regarding themselves during PRB.

## 5. General discussion

This study assessed whether prosocial risky behavior was affected by the interaction between the risk levels of personal cost and the degrees of situational urgency, and it further uncovered the modulation of risk levels of failure when helping others and peer presence in this interaction. We found that compared to non-urgent situations, the weakening effect of the increased risk levels of both personal cost and the failure to help others on social preferences in urgent situations were stronger. Moreover, peer presence can promote PRB among strangers in non-urgent situations involving low-risk levels of personal cost. The results suggest that individuals generally show an obvious altruistic risk-taking tendency during PRB and that both non-urgent situation and peer presence weaken the effect of increased risk levels on PRB to some extent.

### 5.1. Individuals generally show an obvious altruistic risk-seeking tendency when making prosocial risky decisions

This study established that participants involving college students showed an obvious altruistic risk-seeking tendency when making prosocial risky decisions, thereby revealing that participants in risky situations were willing to make more altruistic decisions (than 50% of chance) for strangers in urgent situations. Previous studies have reported that individuals under acute stress make increased altruistic decisions during economic exchange games (Sollberger et al., 2016; Tomova et al., 2017) and when making daily moral decisions (Singer et al., 2017, 2021a,b). For example, people achieve greater procedural and distributive fairness between themselves and strangers regardless of the risk of monetary loss (Brennan et al., 2008; Güth et al., 2008; Karni et al., 2008; Krawczyk and Le Lec, 2010; Gross et al., 2020). Moreover, adolescents show incredible kindness by helping and comforting their peers, family, and strangers in distress (Andrew, 2018). Some recent studies have reported that adolescents demonstrate a remarkable capacity to help others — prosocial risk-taking, and they may do so even when it involves personal risk to their benefits, such as financial, health, academic, or social reputational loss (Do et al., 2017; Blankenstein et al., 2020; Armstrong-Carter et al., 2021). Various studies support the hypothesis that prosocial behavior is linked to an increase in social risk tolerance during adolescence. For example, Armstrong-Carter et al. (2021) found that older adolescents demonstrated increased levels of prosocial tendencies during the years when they were more tolerant of social risk. Furthermore, increased risk levels of both personal cost (Experiment 1) and the failure to help others (Experiment 2) weaken altruistic risk-seeking tendencies. For example, some studies have established that people always show risk aversion when faced with a higher risk level of personal cost, such as monetary loss (Greening et al., 2014), immediate physical painful harm (Hein et al., 2010; Vieira et al., 2020), or hypothetical effective and socially negative outcomes (Saito, 2013; Zhan et al., 2018). Therefore, these findings suggest that prosocial risk-taking tendencies among college students are modulated by the perception of risk at different levels and types.

The theory of reciprocal altruism offered a possible explanation for PRB and held that, although altruistic behavior resulted in some risks of personal cost to the self, it also resulted in immediate or delayed compensations from others. These compensations might be intrinsic motivations and rewards that can improve an individual’s social reputation or standing during social communication (Penner et al., 2005; Dickert et al., 2011). Moreover, another possible mechanism that may account for the increase in the occurrences of both risk-taking and prosocial tendencies during PRB is elevated reward sensitivity (Crone and Dahl, 2012; van Duijvenvoorde et al., 2016). For example, Blankenstein et al. (2020) demonstrated that perspective-taking and intention to comfort uniquely predicted prosocial behavior, whereas fun-seeking predicted both prosocial and rebellious behaviors. Their findings pointed toward a possible differential susceptibility marker, fun-seeking, as a predictor of both prosocial and risk-taking developmental outcomes. Therefore, people in risky or vulnerable situations may be willing to engage in prosocial behaviors for the benefit or social welfare of others despite taking some risks for themselves because that could be driven by a specific type of reward or seeking fun.

### 5.2. Situational urgency modulates the altruistic risk-seeking tendency when making prosocial risky decisions

The results showed that increased risk levels had a more robust weakening effect on altruistic decisions for strangers in urgent situations compared to those in non-urgent situations. This finding was supported by a previous study that reported that more frequent helping decisions under distant (vs. imminent) threats toward strangers would suggest that defensive states associated with the engagement of slower and more flexible decision processes facilitate helping behavior under threats (Buchanan and Preston, 2014). Because situational urgency favors the activation of rapid and reflexive responses, it presumably hinders the engagement of slower and more taxing processes, such as cognitive control and emotional regulation (Mobbs et al., 2007; Qi et al., 2018). These processes may restrain decisions to help others in urgent situations when deciders face a high risk of personal cost. Previous studies have demonstrated that individual differences in sensation-seeking and empathy serve as crucial moderators in the link between social risk perceptions and prosocial tendencies (Schloesser et al., 2013; Do et al., 2017; Blankenstein et al., 2020). In our study, when individuals decide whether to sacrifice self-benefit to help strangers in urgent (vs. non-urgent) situations, they might perceive more urgent situational information and experience stronger emotional and cognitive conflict. Therefore, increased risk levels of personal cost make it easier to mitigate this conflict by reducing helping decisions for strangers in urgent situations. However, this finding was not in accordance with a study showing that regardless of how likely participants were to also receive a shock, they helped the co-participant more under imminent threats than under distal threats (Vieira et al., 2020). We speculate that this difference may be caused by whether the participants were at risk of negative outcomes alone or with others. Increased risk levels of receiving shock, which was shared with others, did not reduce participants’ willingness to help others in urgent situations in Vieria’s study. For example, other studies have also demonstrated that when people share the risk of monetary loss or physical harm with their partners, they are more willing to help their partner despite facing high levels of personal cost (Hein et al., 2010; FeldmanHall et al., 2012; Bixter and Luhmann, 2014). Therefore, in our study, increased risk aversion, which was taken alone, had a more robust weakening effect on helping strangers in urgent (vs. non-urgent) situations. This finding suggests that individuals would weigh and integrate risk and social preferences when making prosocial risky decisions. Specifically, altruistic preferences may have a greater weight influence on PRB in low-level risky helping dilemmas, whereas a tendency toward risk-aversion may have a greater weight influence on PRB in high-risk helping dilemmas.

### 5.3. Different types of risks caused by helping others independently affect prosocial risky behavior

Similar to the risk of personal cost, increased risk levels of failure in helping others also had a more robust weakening effect on helping decisions for strangers in urgent (vs. non-urgent) situations. This finding supports multiple risk perspectives, which argue that multiple risk factors might undermine prosocial developmental trajectories (Evans et al., 2013; Shi et al., 2020). For example, previous studies have reported that increased risk levels of the failure to help others aids people to avoid risk by activating and quickly coordinating specific defense behaviors, such as escape (Fanselow and Lester, 1988; Blanchard and Blanchard, 1990) or indifference to others’ requests for help (Müller and Rau, 2016; Do et al., 2017). However, there was no significant interaction between the two risk levels in Experiment 2. This result was consistent with the assumption of the risk and resilience framework, which was used to evaluate the role of multiple antecedent risk variables (Luthar and Becker, 2000; Cicchetti, 2013). A large body of research suggests that risk factors independently affect prosocial development (Evans et al., 2013; Carlo and Randall, 2014). Shi et al. (2020) pointed out that the independent effects of multiple risk factors on prosocial development are superimposed. Therefore, the second hypothesis that prosocial risk behaviors were synergistically modulated by the levels of the two risks was not supported. We speculated that the effects of risk aversion induced by the two risks on prosocial risk behaviors were superimposed but not mutually restricted. Participants indeed made the least helping frequencies (*M* = 47.75%) in high-risk situations associated with the two risks and the most helping frequencies (*M* = 77.08%) in low-risk situations associated with the two risks. This finding suggests that the combination of the risk of personal cost and the risk of the failure to help others might not significantly enhance individuals’ additional risk aversion but that risk aversion induced by the two risks alone is enough to affect prosocial tendencies.

### 5.4. Peer presence promotes prosocial risky behavior only in dilemmas involving both low-risk and non-urgent situations

Through Experiment 3, we established that individuals in peer presence compared to those in alone situations made more altruistic decisions in dilemmas involving low-level risks of personal cost in non-urgent situations, but not in other risky dilemmas. This finding suggests that peer presence can prompt individuals to engage in increasingly prosocial risky behavior to some extent. However, this promotion is limited to risky dilemmas. This finding is supported by previous studies reporting that peer presence can promote both risk-seeking tendencies (Smith et al., 2014; Tian et al., 2018) and altruistic tendencies (Xin et al., 2016; Wang and Dai, 2020). For example, peer presence can inspire reward or fun seeking, which makes individuals perform better by engaging in more risky or altruistic behaviors for others to improve their social reputation and standing in the community (Cavalca et al., 2013; Chase et al., 2017). However, in our study, peer presence can prompt college students to make more helpful decisions only in these relatively safer dilemmas, where they are faced with a lower risk of personal cost and experience non-urgent information and weak emotional conflict. This finding was supported by the life-history perspective, which indicated that life stress caused by peer presence is associated with lower risk aversion and lower present orientation (Giudice et al., 2011; Nettle, 2016). Peer presence predicts that individuals’ reputations are in dangerous environments (Rickard et al., 2014) and that they might affect risk and social preferences in ways that facilitate their strategy and cost-effectiveness (Griskevicius et al., 2013; Wu et al., 2020). Specifically, individuals valued both immediate benefits and long-term consequences, and therefore, they took a safer approach (e.g., low-risk levels of personal cost and weak internal conflict) to maintain their reputations (Figueredo et al., 2006; Gilbert and Basran, 2019). Therefore, this finding suggests that the promotion effect of peer presence on PRB is modulated by both the risk levels of personal cost and situational urgency.

### 5.5. Limitations and directions for future research

This study has some limitations. First, although our study provides some preliminary causal evidence for the interaction between risk and social preferences in PRB, it is not yet possible to quantify the switching point of risk aversion. Future studies should focus on the gain-loss matrices between the self and others in risky helping dilemmas involving multiple risk levels of personal cost. Second, while it was beyond the scope of our study to examine individual differences, i.e., sensation-seeking, social value orientation, or empathy, in the trade-off and integration between risk and prosocial preferences, future studies should explore this possibility because PRB is thought to be related to individuals’ sensation-seeking and empathy abilities (Blankenstein et al., 2020; Armstrong-Carter et al., 2021; McGowan et al., 2022). Third, G* Power 3.1 software does not calculate the minimum number of subjects needed to generate interactions between variables within groups, except for the between-group effects and interactions that are the focus of this study. Future studies should be pre-registered and the sample size further expanded to ensure that the studies have rigorous statistical power to test. Finally, the samples used in this study only involved Chinese college students, and they lacked participants from other age groups, especially adolescents. Future studies should provide more causal evidence of the trade-offs between risk and social preferences among adolescents.

## 6. Conclusion

This study aimed to adopt the risky helping dilemma task to provide causal evidence of the interaction between risk levels and situational urgency associated with prosocial risky behaviors and further explore the modulation of risk types and peer presence. The results showed that individuals generally showed an obvious risk-taking tendency during prosocial risky behaviors, whereas the increased risk levels of both personal cost and the failure to help others had a stronger weakening effect on the frequencies of help provided to strangers in urgent situations relative to those in non-urgent situations. Furthermore, peer presence encouraged participants to make more altruistic choices toward strangers in non-urgent situations. However, this promotion effect was observed only in situations involving low-risk levels of personal cost. Our study provides novel and empirical research perspectives for investigating the mechanisms behind the trade-off and integration between risk and social preferences when making prosocial risky decisions and behaviors in risky and vulnerable contexts. Altogether, the findings suggest that individuals generally show an obvious altruistic risk-seeking tendency when making prosocial risky decisions and that both non-urgent situations and peer presence weaken the effect of increasing risk aversion on PRB to some extent.

## Data availability statement

The original contributions presented in the study are included in the article/Supplementary material, further inquiries can be directed to the corresponding author.

## Ethics statement

The studies involving human participants were reviewed and approved by the ethics committee of Hunan University of Science and Technology. The patients/participants provided their written informed consent to participate in this study.

## Author contributions

CL and XX prepared the materials and measures. CL and QP collected and analyzed the data. CL and QT drawn tables and figures. CL wrote and amended the manuscript. YZ reviewed the manuscript. All authors contributed to the article and approved the submitted version.

## Funding

This work was supported in part by grants from the Natural Science Foundation of Hunan Province (2021JJ40196), the Key Project of the Philosophy Social Science Project of Hunan Province (18ZDB011), the Educational Science Planning Program of Hunan Province (XJK19QXL002), the Project of Social Science Evaluation Committee of Hunan Province (XSP20YBZ030 and XSP19YBZ082), and the Postgraduate Scientific Research Innovation Project of Hunan Province (CX20211014).

## Conflict of interest

The authors declare that the research was conducted in the absence of any commercial or financial relationships that could be construed as a potential conflict of interest.

## Publisher’s note

All claims expressed in this article are solely those of the authors and do not necessarily represent those of their affiliated organizations, or those of the publisher, the editors and the reviewers. Any product that may be evaluated in this article, or claim that may be made by its manufacturer, is not guaranteed or endorsed by the publisher.
