# Supplementary material for: Are you more risk-seeking when helping others? Effects of situational urgency and peer presence on prosocial risky behavior
Source: Front Psychol. 2023 Feb 27;14:1036624. doi: 10.3389/fpsyg.2023.1036624 (PMC10020997; doi:10.3389/fpsyg.2023.1036624)
Supplement: Supplementary file 1 [file Table_1.docx]

**Helping Dilemmas**

**Urgent situations:**

1. **When you are on the way to the postgraduate entrance examination, someone is hit by a car. He is in dire need of your help to send him to the hospital for treatment.**

F: If you help, he will get medical attention, but you will be X likely to miss the exam.

J: Without help, he will be in danger of not being treated in time, but you will take the exam successfully.

1. **Someone embezzled public funds, you check the accounts after the discovery, request you do not report, because his father is in urgent need of money for surgery, and promise that the next check before the loopholes will be filled.**

F: If you help, his father will get medical attention, but you will be X likely to be criminally punished.

J: Without help, he will be punished and his father will not receive prompt medical attention, but you will not be punished.

1. **Someone is being held hostage by a robber who is robbing a jewelry store. You are a salesman in the jewelry store. You rang the alarm in time, but the robber jumped the wall and took him hostage.**

F: If you help, his life is not in danger, but you will be X likely to get reprisals from a gangster.

J: Without help, his life will be in danger, but you can keep yourself safe.

1. **A man who has survived a shipwreck comes upon your ship, which has just escaped, and which is now full, and is on the surface of the water asking for help from your captain.**

F: If you help, he will board your ship, but there is a X chance that your ship will sink.

J: Without help, he will not have the strength to wait for the next rescue, but yours will land safely.

1. **Someone was seriously injured by the explosion of liquefied gas at home. You are a taxi driver. His family took him in your car to the hospital. In order to save time for rescue, he asked you to run the red light to save time.**

F: If you help, he will get immediate medical attention, but you will be X likely to lose your license and your job.

J: Without help, he will die from delayed treatment, but you will not be held accountable by traffic control.

1. **Someone had a heart attack on the bus. Today is your first day at work. You haven't waited for the bus. The time is very urgent, but he took out the money and asked you to take him to the hospital.**

F: If you help, he will get immediate medical attention, but you will be X likely to be late for work and lose a job you love.

J: Without help, his life will be in danger, but you will be successful in your favorite job

1. **Someone fell to the ground in front of the exhibition hall because of a car accident. The driver has escaped. You are a venue volunteer. He asks you to send him to a nearby hospital for treatment.**

F: If you help, he will be treated in time, but you will be X likely not to be able to complete your work in time and there will be a serious accident at the exhibition.

J: Without help, he will lose his life due to delayed treatment, but your work will not be affected.

1. **Someone is robbed on the road. You happen to pass by. He cries out to you for help.**

F: If you help, he will be saved, but you will be X likely to get reprisals.

J: Without help, he will lose property and be hurt, but your safety will be guaranteed.

1. **Someone has a flat tire on the highway and there is a pregnant woman waiting to give birth in the car. At this time, you are rushing back to your hometown to visit your seriously ill relatives. Just as you pass by, he waves to you and asks for help.**

F: If you help, the pregnant woman will have a smooth delivery, but you will be X likely not to see your loved one last.

J: Without help, the pregnant woman will die, but you will make it home in time.

1. **Someone will die due to illness. The ambulance is taking him to the hospital. You are a fire truck driver who is giving out a fire. At this time, the ambulance whistles to ask you to overtake and pilot and arrive at the hospital as soon as possible.**

F: If you help, the patient will be treated, but you will be X likely to miss work and cause a serious fire.

J: Without help, he will die of delayed treatment, but the fire will be put out in time.

**Non-urgent situations:**

1. **Someone is a poor student in the village, who is in urgent need of a large number of tuition fees after he is admitted to the university. You are a poverty alleviation village cadre. The village has just applied for a sum of money to repair the well, and the villagers are excited, but at this time he asks you for help.**

F: If you help, his tuition problem will be solved, but you will be X likely to delay the well repair work and be blamed by the villagers.

J: Without help, he will not be able to go to school and thus abandon his studies, but the task will be completed successfully.

1. **Someone's leg was injured in the process of climbing. At this time, you were climbing alone and found him. He asked you to help him down the mountain to the nearby hospital for medical treatment.**

F: If you help, his leg will be saved, but you will be X likely to lose your expensive climbing gear.

J: Without help, his leg will have a hangover, but you will not lose anything.

1. **Someone was knocked down by a motorcycle at night and his leg was injured. You are a small vendor who sells fruit for a living. You have to set up a stall regularly every day for a family of four. At this time, he asks you to send him to a nearby hospital for immediate treatment.**

F: If you help, his leg will be saved, but you will be X likely not to make up for the loss.

J: Without help, his leg will have a hangover, but you will not suffer economic losses.

1. **Someone’s finger bled after being bitten by a dog. You are a head teacher of primary school students. You are taking children on a spring outing. There are no other adults nearby. He asks you to send him to a nearby hospital for treatment.**

F: If you help, his finger will be bandaged in time, but you will be X likely to cause an accident to a child.

J: Without help, his finger will have a hangover, but children's safety is guaranteed.

1. **Someone has a sudden high blood pressure and faints in the supermarket late at night. You are a supermarket salesperson on duty alone. Are you willing to send him to the hospital for first aid.**

F: If you help, he will be saved, but you will be X likely to lose your job.

J: Without help, he will miss the best treatment time and have a hangover, but you will not be held accountable.

1. **Someone has a mental illness to seek medical treatment. You are a famous psychological counselor. It is found that this person has committed a crime during the meeting. He asks you not to expose it to the police.**

F: If you help, his crimes will not be uncovered, but you will be X likely to be held accountable by the police.

J: Without help, he will be arrested by the police, but you will not be punished by the law.

1. **Someone is suffering from a chronic disease and his family is poor. You are a pharmacist in a research institute. He asks you to sell this expensive medicine to him at a low price without permission.**

F: If you help, his financial difficulties are solved, but you will be X likely to lose your job for violating company policy.

J: Without help, he will be sick, but you will not be held accountable.

1. **Someone has gastroenteritis and still needs some treatment expenses. You and he are co-workers. They have just received half a year's salary and plan to send the living expenses for the second half of the year to their wife and children, but he really can't ask you to borrow money.**

F: If you help, his gastroenteritis is alleviating, but he will be X likely not to pay you back on time and your wife and children will not have enough to live on.

J: Without help, he will suffer from diarrhea, but the normal living expenses of your wife and children will be guaranteed.

1. **Someone needs to pay the credit card bill. You and he are classmates. They just got the living expenses of next month. Now they borrow money from you.**

F: If you help, he will not have to pay overdue interest on his bank card, but he will be X likely not to pay you back on time and you will not have enough to live on.

J: Without help, he will have to pay overdue interest on his bank card, but the normal living expenses of yourself will be guaranteed.

1. **Someone is anxiously waiting for a taxi by the side of the road. At this time, you are driving to a hospital to see your wife in labor. He asks you to take him to the station so as not to miss the train.**

F: If you help, he will ride the train, you will be X likely not to be with your wife in labor.

J: Without help, he will not arrive at the station on time, but you will witness the birth of your child.
